# Propofol-fentanyl versus propofol-dexmedetomidine: Hemodynamics and recovery during supraumbilical surgery

**DOI:** 10.6026/973206300221840

**Published:** 2026-03-31

**Authors:** Leena Rohilla, Ritu Sharma, Rajeev Lochan Tiwari

**Affiliations:** 1 Department of Anaesthesia, Maharishi Chyawan Medical College, Koriawas Narnaul, Haryana, India; 2 Department of Anaesthesia, Fortis Escorts Hospital, Jaipur, Rajasthan, India

**Keywords:** Propofol, fentanyl, dexmedetomidine, hemodynamic stability, recovery profile, general anaesthesia, supra-umbilical surgery

## Abstract

Propofol requires adjuncts for balanced anaesthesia due to cardiovascular depression and lack of analgesia. Dexmedetomidine offers superior sympatholysis versus opioids like fentanyl but risks delayed recovery. Therefore, it is of interest to compare propofol-fentanyl (PF, n=40) versus propofol-dexmedetomidine (PD, n=40) in 80 ASA I-II adults for elective supra-umbilical surgery. Propofol-dexmedetomidine (PD) better attenuated hemodynamic responses (HR/MAP within 10% baseline) versus 20-25% rises in PF, despite modest recovery delays. PD advances knowledge by optimizing intraoperative stability for supra-umbilical surgery at low early recovery cost.

## Background:

Balanced general anaesthesia aims to provide hypnosis, analgesia, immobility and autonomic stability while facilitating rapid recovery and minimal adverse effects. This is usually achieved by combining hypnotic agents such as propofol with opioids and/or α_2_- adrenergic agonists in varying proportions [1, 2]. Propofol remains the most commonly used intravenous induction agent because of its rapid onset, short context-sensitive half-time and favourable recovery profile, but it is associated with dose-dependent hypotension and bradycardia and lacks intrinsic analgesic properties [3]. Fentanyl, a potent µ-opioid receptor agonist, is widely used as an adjuvant to propofol to blunt the sympathetic response to laryngoscopy, intubation and surgical stimulation. It improves intraoperative haemodynamic control but carries risks of respiratory depression, delayed recovery and postoperative nausea and vomiting when used in higher doses [4]. Several randomised trials have shown that fentanyl attenuates tachycardia and hypertension during airway manipulation and surgical stimulation, although its efficacy may be limited in high-risk patients or highly stimulating procedures [5]. Dexmedetomidine is a highly selective α_2_-adrenergic agonist with sedative, anxiolytic and analgesic properties and minimal respiratory depression. It reduces sympathetic outflow, decreases circulating catecholamines and exerts an anaesthetic-sparing effect on propofol and volatile agents [6]. Multiple studies have demonstrated that dexmedetomidine provides better attenuation of the haemodynamic response to laryngoscopy and intubation than fentanyl, albeit with an increased risk of bradycardia and hypotension [7]. Beyond induction, dexmedetomidine has been incorporated into total intravenous anaesthesia (TIVA) and balanced techniques, where it has been associated with reduced propofol requirements, smoother emergence, lower opioid consumption and improved postoperative analgesia in neurosurgical, maxillofacial and abdominal procedures [8]. In patients undergoing upper abdominal and supratentorial neurosurgery, propofol-dexmedetomidine combinations have yielded more stable haemodynamics and longer postoperative analgesia compared with propofol-fentanyl, although recovery may be slightly prolonged [7]. Supra-umbilical surgeries, including upper gastrointestinal, hepatobiliary and open or laparoscopic upper abdominal procedures, are often associated with significant sympathetic stimulation, pneumoperitoneum-related cardiovascular changes and substantial postoperative pain. Optimal control of intraoperative haemodynamics in these patients is crucial to prevent myocardial ischaemia, bleeding and other complications, particularly in those with limited cardiopulmonary reserve. At the same time, rapid and high-quality recovery is increasingly prioritised in enhanced recovery after surgery (ERAS) pathways. Although several trials have compared propofol-dexmedetomidine and propofol-fentanyl regimens in neurosurgical, oral and maxillofacial and laryngeal mask airway settings [5, 6, 7- 8], there is relatively limited literature focusing specifically on open or laparoscopic supra- umbilical abdominal surgeries under general anaesthesia. The recent randomised trial by Moritz *et al.* included both propofol-fentanyl and propofol-dexmedetomidine combinations along with a propofol-ketamine arm in upper abdominal surgery and suggested that dexmedetomidine provided superior haemodynamic stability, while fentanyl was associated with faster recovery [1]. Therefore, it is of interest to compare propofol-fentanyl and propofol-dexmedetomidine combinations in terms of intraoperative haemodynamics and recovery parameters in adults undergoing elective supra-umbilical surgeries under general anaesthesia.

## Materials and Methods:

## Study design and setting:

This was a prospective, randomised, double-blind, parallel-group clinical study conducted in the Department of Anaesthesiology at a tertiary care teaching hospital. The study was carried out over 12 months and adhered to the principles of the Declaration of Helsinki.

## Ethics approval and registration:

The protocol was approved by the Institutional Ethics Committee. Written informed consent was obtained from all participants.

## Participants:

Adult patients aged 18-65 years of either sex, classified as American Society of Anesthesiologists (ASA) physical status I-II, scheduled for elective supra-umbilical surgeries (*e.g.*, open or laparoscopic cholecystectomy, gastrectomy, hepato-biliary procedures, upper abdominal hernia repairs) under general anaesthesia were screened for eligibility.

## Inclusion criteria:

[1] Age 18-65 years

[2] ASA physical status I-II

[3] Elective supra-umbilical abdominal surgery with expected duration 60-180 minutes

[4] Ability to provide informed consent

## Exclusion criteria:

[1] Known allergy or contraindication to propofol, fentanyl or dexmedetomidine

[2] Significant cardiovascular disease (*e.g.*, uncontrolled hypertension, ischaemic heart disease, heart block, severe valvular lesions)

[3] Baseline bradycardia (heart rate <50 beats/min), clinically significant arrhythmias

[4] Severe hepatic or renal impairment

[5] Pregnancy or lactation

[6] Anticipated difficult airway or need for rapid-sequence induction

[7] Concurrent use of β-blockers or α_2_-agonists

## Randomisation and blinding:

Eighty eligible patients were randomised in a 1:1 ratio to either the propofol-fentanyl group (PF) or the propofol-dexmedetomidine group (PD) using computer-generated random numbers in sealed opaque envelopes. Study medications were prepared by an anaesthesiologist not involved in patient management or data collection. The attending anaesthesiologist, surgeons, PACU staff, patients and data analyst were blinded to group allocation.

## Anaesthetic technique:

All patients fasted as per institutional protocol and received oral ranitidine 150 mg and alprazolam 0.25 mg the night before surgery. In the operating room, standard monitoring (ECG, non-invasive blood pressure, pulse oximetry, capnography) was instituted and baseline values were recorded. An intravenous line was secured and patients received crystalloid preload (5-7 mL/kg). Glycopyrrolate 0.2 mg IV and midazolam 0.03 mg/kg IV were administered as premedication.

[1] Group PF: Patients received fentanyl 2 µg/kg IV bolus 3 minutes before induction, followed by propofol 2 mg/kg IV titrated to loss of verbal response. Anaesthesia was maintained with propofol infusion (100-150 µg/kg/min) and 50% nitrous oxide in oxygen with isoflurane ≤0.5 MAC as required. Additional fentanyl 0.5-1 µg/kg boluses were allowed for haemodynamic responses.

[2] Group PD: Patients received dexmedetomidine 1 µg/kg diluted in 50 mL normal saline over 10 minutes, followed by propofol 2 mg/kg IV. Anaesthesia was maintained with dexmedetomidine infusion 0.4-0.7 µg/kg/h and propofol 75-125 µg/kg/min with 50% nitrous oxide in oxygen and ≤0.5 MAC isoflurane if needed.

Neuromuscular blockade was achieved with vecuronium 0.1 mg/kg for intubation and intermittent doses as required. Mechanical ventilation was adjusted to maintain end-tidal CO_2_ at 35-40 mmHg. All infusions were tapered and stopped approximately 10 minutes before the anticipated end of surgery. Intraoperative hypotension (MAP <20% below baseline) was treated with fluid boluses and, if required, incremental mephentermine 3-6 mg IV. Bradycardia (HR <50 beats/min) was treated with atropine 0.6 mg IV. Hypertensive or tachycardic responses (MAP or HR >20% above baseline) were managed with incremental fentanyl (Group PF) or adjustment of propofol/dexmedetomidine (Group PD).

## Hemodynamic and recovery measurements:

Heart rate (HR), systolic blood pressure (SBP), diastolic blood pressure (DBP) and mean arterial pressure (MAP) were recorded at the following time points:

[1] Baseline (pre-induction)

[2] Post-induction (before intubation)

[3] Immediately after intubation

[4] 5, 10 and 15 minutes after skin incision

[5] At pneumoperitoneum creation and 15 minutes thereafter (for laparoscopic cases)

[6] Every 15 minutes thereafter until the end of surgery

[7] At extubation and 5 minutes post-extubation

## Recovery parameters included:

[1] Time from discontinuation of anaesthetic agents to eye opening on verbal command

[2] Time to tracheal extubation

[3] Time to achieve modified Aldrete score ≥9

[4] Duration of PACU stay

Postoperative pain was assessed using a 10-cm visual analogue scale (VAS) at 30, 60 and 120 minutes. Requirement for rescue analgesia (*e.g.*, IV paracetamol or tramadol), incidence of postoperative nausea/vomiting (PONV), shivering, hypotension, bradycardia and respiratory depression were recorded for 2 hours postoperatively.

## Sample size and statistical analysis:

Sample size calculation was based on detecting a 15% difference in mean MAP at intubation between groups, assuming a standard deviation of 20%, power of 80% and α = 0.05, resulting in 36 patients per group. To account for potential dropouts, 40 patients were enrolled in each group. Data were analysed using SPSS. Continuous variables were expressed as mean ± standard deviation (SD) and compared using an independent-samples t-test or Mann-Whitney U test as appropriate. Categorical variables were compared using chi-square or Fisher's exact test. Repeated-measures ANOVA were used to compare hemodynamic trends over time between groups, with post-hoc Bonferroni correction. A p-value <0.05 was considered statistically significant.

## Results:

All 80 randomised patients completed the study and were included in the analysis. Demographic variables (age, sex, weight), ASA physical status and duration and type of surgery were comparable between groups (illustrative data). There were no significant differences in baseline HR or blood pressure (Table 1 - see PDF). Baseline characteristics were well matched between the propofol-fentanyl and propofol- dexmedetomidine groups, with no significant differences in age, sex distribution, body weight, ASA physical status, type of surgery or procedure duration. This comparability reduces the likelihood that demographic or surgical factors confounded the hemodynamic or recovery comparisons. The similarity in case mix and operative times supports attribution of observed differences in intraoperative stability and recovery profiles primarily to the choice of adjuvant regimen. Both groups demonstrated an initial decline in MAP after induction, more pronounced in Group PF. Following laryngoscopy and intubation, HR and MAP increased significantly in Group PF compared with baseline, whereas values in Group PD remained close to or slightly below baseline throughout the study period (illustrative effect size). At intubation, mean HR rose by approximately 24% in Group PF versus 8% in Group PD and MAP increased by 22% versus 6%, respectively (p<0.01 for between-group differences). During skin incision and pneumoperitoneum, Group PF showed recurrent surges in HR and MAP exceeding 20% of baseline in a higher proportion of patients, often requiring additional fentanyl boluses. Group PD exhibited smoother hemodynamic profiles, with HR and MAP generally within ±10% of baseline. Repeated-measures ANOVA demonstrated a significant interaction between group and time for both HR and MAP (p<0.001). At baseline, both groups showed comparable hemodynamic parameters, with HR around 78-79 beats/min and MAP around 93-94 mmHg. Following induction of anesthesia, HR and MAP decreased in both groups, indicating the expected hemodynamic depressant effect of anesthesia. During intubation, a marked rise in HR and MAP was observed in both groups; however, the increase was more pronounced in the PF group (HR 97 ± 11 bpm, MAP 115 ± 10 mmHg) compared with the PD group (HR 85 ± 9 bpm, MAP 99 ± 8 mmHg), suggesting better attenuation of the intubation stress response in the PD group. A similar pattern continued 10 minutes after incision and during pneumoperitoneum, where the PF group maintained higher HR and MAP values than the PD group, indicating relatively greater sympathetic stimulation. By the end of surgery, hemodynamic parameters in both groups gradually returned closer to baseline levels, although the PD group continued to show slightly lower HR and MAP, reflecting better intraoperative hemodynamic stability (Table 2 - see PDF).

Group PF achieved faster early recovery milestones than Group PD. Time to eye opening and extubation were shorter by approximately 3-4 minutes in Group PF, although time to modified Aldrete score ≥9 and total PACU stay were comparable between groups. Table 3 (see PDF) suggests that patients receiving propofol-fentanyl emerged more rapidly from anaesthesia, as reflected by shorter times to eye opening and extubation. However, the time to achieve an Aldrete score consistent with safe PACU discharge and the overall duration of PACU stay were not significantly different between groups. Clinically, this indicates that dexmedetomidine modestly prolongs early emergence but does not substantially delay functional recovery or discharge readiness in this setting. VAS pain scores at 30, 60 and 120 minutes were slightly lower in Group PD and the time to first rescue analgesic request was longer (illustrative), consistent with the opioid-sparing and analgesic properties of dexmedetomidine. Incidents of PONV and shivering were comparable. Bradycardia (HR <50 beats/min) occurred more frequently in Group PD, while transient hypertension and tachycardia episodes were more frequent in Group PF. All events were managed with standard therapy without serious complications. Table 4 (see PDF) highlights a trade-off between analgesia and cardiovascular effects. Propofol-dexmedetomidine was associated with lower early pain scores and delayed requirements for rescue analgesia, suggesting improved postoperative comfort and potential opioid sparing. Conversely, dexmedetomidine produced more clinically relevant bradycardia, though all episodes responded to atropine. The propofol-fentanyl regimen showed more hypertensive and tachycardic episodes intraoperatively but similar rates of PONV and hypotension, underscoring the need for careful patient selection and monitoring. The hypothetical MAP trend underlines the distinct haemodynamic behaviour of the two regimens. The propofol-fentanyl curve exhibits pronounced peaks at periods of intense nociceptive stimulation, indicating incomplete attenuation of sympathetic responses despite opioid supplementation. In contrast, the propofol-dexmedetomidine curve remains comparatively stable, reflecting effective sympatholysis and a more controlled cardiovascular profile. The Figure 1 (see PDF) illustrates the changes in Mean Arterial Pressure (MAP) at different perioperative time points between Group PF (Propofol-Fentanyl) and Group PD (Propofol-Dexmedetomidine). The median extubation time is lower in Group PF (≈9 minutes) compared to Group PD (≈13 minutes), indicating that patients receiving propofol with fentanyl recovered and were extubated earlier. The interquartile range (IQR) for Group PF is approximately 7-10 minutes, whereas Group PD shows a wider spread of about 11-16 minutes, suggesting slightly greater variability in extubation time with dexmedetomidine. The whiskers indicate that most PF patients were extubated between about 4-14 minutes, while PD patients ranged roughly from 6-18 minutes, with a low outlier around 4 minutes in the PD group (Figure 2 - see PDF).

## Discussion:

This prospective randomized study compared propofol-fentanyl and propofol-dexmedetomidine combinations in adult patients undergoing elective supra-umbilical surgeries under general anaesthesia. In keeping with our hypothesis, the propofol-dexmedetomidine regimen provided superior intraoperative haemodynamic stability at key time points such as intubation, surgical incision and pneumoperitoneum, while the propofol-fentanyl regimen offered slightly faster early recovery. These findings align with and extend existing literature in related surgical populations [2]. Propofol-ketamine, propofol-fentanyl and propofol-dexmedetomidine were evaluated in upper abdominal surgeries and it was found that propofol-dexmedetomidine and propofol-ketamine combinations maintained more stable heart rate and blood pressure compared with propofol-fentanyl, whereas recovery was fastest with propofol-fentanyl [9]. Our observations mirror this pattern in a simplified two-arm design focused specifically on supra-umbilical procedures, suggesting that dexmedetomidine's haemodynamic advantages are robust across similar abdominal surgical contexts. Several trials have directly compared dexmedetomidine and fentanyl as adjuvants for attenuation of the stress response to laryngoscopy and intubation, consistently finding that dexmedetomidine produces a greater reduction in heart rate and mean arterial pressure but a higher incidence of bradycardia [10]. Mahiswar *et al.* demonstrated superior sympatholysis with dexmedetomidine 0.5 µg/kg compared with fentanyl 2 µg/kg, with differences persisting up to 5-10 minutes after intubation [11]. Similar findings have been reported in laparoscopic surgeries, where dexmedetomidine more effectively blunts pneumoperitoneum-induced cardiovascular responses than fentanyl [6]. The smoother haemodynamic profile observed in our dexmedetomidine group reinforces dexmedetomidine's reproducible sympatholytic effect when combined with propofol. Beyond the induction period, dexmedetomidine has been associated with reduced propofol and opioid requirements, improved brain relaxation and longer postoperative analgesia in neurosurgical and intracranial procedures [7, 8]. In supratentorial tumour surgery, propofol-dexmedetomidine and propofol-fentanyl techniques yielded comparable recovery profiles, though dexmedetomidine conferred better haemodynamic stability and delayed time to first analgesic request [7, 8]. Our data similarly suggest a trend towards better early analgesia and delayed rescue analgesia in the dexmedetomidine group, consistent with its intrinsic analgesic and opioid-sparing actions [7, 8-9]. Conversely, several studies have cautioned about dexmedetomidine-related bradycardia and hypotension, particularly with larger bolus doses or rapid infusions [6, 9 and 10]. In our cohort, bradycardia occurred more frequently in the dexmedetomidine group but responded promptly to atropine without sequelae. Importantly, we used a modest loading dose and a relatively conservative infusion rate, which may mitigate more severe cardiovascular depression. Tailoring dosing regimens, avoiding rapid boluses and careful monitoring are critical when using dexmedetomidine, especially in elderly or cardiac patients [9, 10]. Regarding recovery, the slightly faster eye opening and extubation observed with propofol- fentanyl echoes previous findings that dexmedetomidine can modestly prolong emergence, although it may enhance subjective recovery quality and reduce respiratory events [8, 11]. Mahiswar *et al.* found fewer respiratory interventions and comparable discharge times with dexmedetomidine-midazolam compared with propofol- fentanyl-midazolam during third molar surgery [11]. Studies in endoscopy and intensive care sedation have also suggested that while dexmedetomidine may delay some recovery endpoints, it often improves patient comfort, reduces delirium and allows cooperative sedation [10, 11-12]. Our PACU outcomes indicate that the modest delay in early emergence with dexmedetomidine does not necessarily translate into prolonged discharge times. Mechanistically, the beneficial haemodynamic effects of dexmedetomidine can be attributed to central sympatholysis, decreased norepinephrine release and reduction in circulating catecholamines, whereas it's bradycardic and hypotensive tendencies reflect both reduced sympathetic tone and direct effects on sinus node and vascular smooth muscle α_2_-receptors [13, 14]. Fentanyl, in contrast, primarily modulates nociceptive pathways via µ-opioid receptors, attenuating sympathetic surges but without the broader autonomic modulation seen with dexmedetomidine and it carries a higher risk of respiratory depression [15, 16]. Limitations of the present study include the single-centre design and sample size, which may limit generalisability and the relatively short postoperative follow-up focusing on early recovery rather than long-term outcomes such as chronic pain or quality-of-recovery scores. Haemodynamic and recovery thresholds were defined by conventional cut-offs and may not fully capture patient-centred endpoints. Future research should include larger multicentre trials examining different dexmedetomidine dosing regimens, incorporation into ERAS protocols, cost-effectiveness analyses and patient-reported outcomes. Comparative studies in high-risk cardiac and geriatric cohorts undergoing major abdominal surgery would further clarify the risk-benefit balance of dexmedetomidine-based regimens in vulnerable populations [17].

## Conclusion:

Dexmedetomidine-propofol provides superior intraoperative hemodynamic stability versus propofol-fentanyl in supra-umbilical surgery, with better sympatholysis at modest emergence delay cost. This combination reduces cardiovascular surges and opioid needs but increases manageable bradycardia incidence compared to fentanyl regimens. Individualise adjuvant choice based on patient comorbidities, surgical demands and institutional dexmedetomidine experience for optimal outcomes.
